# Applicability of handheld laser scanning for nonmetric skeletal assessment in biological profile estimation: a preliminary two-case study

**DOI:** 10.1007/s00414-026-03943-6

**Published:** 2026-08-04

**Authors:** Chloe R. Cummings, Emily N. Powell, An-Di Yim

**Affiliations:** 1 Forensic Science Program, George Mason University, Fairfax, VA USA; 2 Department of Anthropology, University of Illinois Urbana-Champaign, Urbana, IL USA

**Keywords:** Forensic anthropology, Three-dimensional (3D) models, Surface scanning, Inter-observer reliability, Virtual anthropology, Nonmetric traits

## Abstract

**Background:**

In forensic anthropology, skeletal materials are commonly assessed through morphological characteristics to estimate components of the biological profile. While traditional analyses relied on physical examination of skeletal remains, digital methodologies offer the potential to supplement or replace direct observations, increasing the accessibility of forensic anthropological consultation, research, and education. However, the reliability of three-dimensional (3D) documentation techniques for nonmetric assessment remained variable and required further investigation. This study provides a preliminary investigation of the applicability of handheld laser scan-derived point-cloud models for nonmetric skeletal assessment in biological profile estimation using a two-case design.

**Materials and Methods:**

Two skeletal cases were selected from the George Mason University Forensic Science Program Teaching Collection: a 40-year-old White male and an 80-year-old White female. A total of 53 participants with varying levels of forensic anthropological training and experience were recruited from the Washington metropolitan area. Participants first conducted in-person assessments of skeletal elements and subsequently evaluated 3D models after a washout period.

**Results:**

Inter-observer agreement was generally higher for physical skeletal assessments than for virtual assessments. Skeletal sex estimation (particularly innominate traits) and age estimation via the pubic symphysis showed lower agreement and reduced inter-modality correspondence. Despite this, skeletal sex classification accuracy remained largely stable across modalities. Conversely, population affinity, despite fair inter-modality agreement, showed reduced classification accuracy in the virtual setting.

**Conclusion:**

These findings suggest that handheld laser scans do not fully replicate all aspects of nonmetric skeletal assessment, and their application should be considered trait-dependent and validated further before routine use.

**Supplementary Information:**

The online version contains supplementary material available at https://doi.org/10.1007/s00414-026-03943-6.

## Introduction

Forensic anthropologists are often tasked with the examination of highly decomposed or skeletonized human remains [1, 2]. This typically includes an analysis of skeletal material using both morphological and metric data to build a biological profile, consisting of sex, population affinity, age, and stature [1–3]. Traditionally, these analyses are performed by direct examination, which can contribute to wear and handling-related damage over repeated use, particularly in research and teaching collections [4, 5].

Digital imaging can be used to supplement or replace these direct observations [3, 6–8]. These methods include three-dimensional (3D) imaging approaches such as computed tomography (CT) [8–11], 3D photogrammetry [3], and laser surface scanning [6], and they have become increasingly integrated into biological and forensic anthropology for documentation, morphometric analysis, virtual curation, and remote consultation [9, 12]. The most commonly used surface scanners are 3D laser and structured light systems. These systems record the external surface of an object by providing a 3D topographic data set [7, 13], generating point cloud data points denoting precise 3D geometric locations recorded on the surface of an object, which can be inspected directly or converted into polygonal and triangular mesh formats to create a shape with a continuous 3D surface [7, 13, 14].

FARO^®^ scanners are a type of laser scanning system that has been widely used for crime scene documentation. The Freestyle system, in particular, is a family of lightweight, handheld scanners that allow for additional mobility and utility when operating and navigating crime scenes [15]. The effectiveness of this investigative tool has been assessed for use on blood stain patterns, fingerprints, broken glass, two-dimensional (2D) and 3D footwear impressions, and deriving text from written documents [15]. When applied to metric skeletal characteristics, Mullins and Albanese [16] showed that, while there is a tendency for slight measurement error (mean deviation of less than 3.03 mm, with a tendency to undermeasure), the general size of the scanned skeletal elements was similar to the actual objects. Their results indicate that such handheld laser scanning system can be used to provide relatively accurate measurements and establish a faithful recreation of a skeletal element. However, the applicability of laser scanning systems such as the FARO^®^ Freestyle to document nonmetric skeletal characteristics remains unknown.

Moreover, although more specialized imaging systems have been shown to be effective regarding forensic anthropological interpretations [3, 6–8, 10, 11], more general forensic capturing methods, like the FARO^®^ system, have yet to be extensively evaluated. In addition, results for the reliability of 3D scanning techniques are mixed within the literature and warrant further investigation [6, 16–19]. The purpose of this study is to evaluate the applicability of handheld laser scan-derived 3D skeletal models for nonmetric biological profile estimation in forensic anthropology. Specifically, this study assessed whether trait scoring and biological profile estimation derived from virtual models differed from those obtained through direct examination of skeletal remains.

## Materials and methods

Two skeletal cases were selected from the George Mason University Forensic Science Program Teaching Collection. Both cases are individuals who consented to body donation to the Forensic Science Research and Training Laboratory via the Virginia State Anatomical Program (VSAP), a division of the Commonwealth of Virginia’s Department of Health. Case 1 was a 40-year-old White male, and case 2 was an 80-year-old White female. These two cases were selected to capture a broad range of nonmetric traits: both skeletal sexes are represented, the difference in age between the two cases provides different morphological details in age traits, and while both individuals are reported to be White, Case 2 possesses more ambiguous nonmetric characteristics. Only the skulls and innominates were used for this study. The reference trait scores and biological profile estimation results were provided in the Supplementary Information (Table S1).

The FARO^®^ Freestyle 2 handheld scanner (FARO Technologies, Inc., Lake Mary, FL) was used to generate 3D virtual models of the skeletal elements. Each element was scanned from a three-foot distance, using plane detection, color scan, flash engaged, and with white balance applied to the scan [20]. Each element was captured in two or three scans in both anterior and inferior views to ensure that all surfaces of the elements were captured. FARO^®^ Circular Scan markers were used to ensure the scans were able to be registered. The resulting scans were then imported into the proprietary FARO^®^ SCENE software [20], where they were manually registered, rendered, and cleaned.

**Fig. 1:**
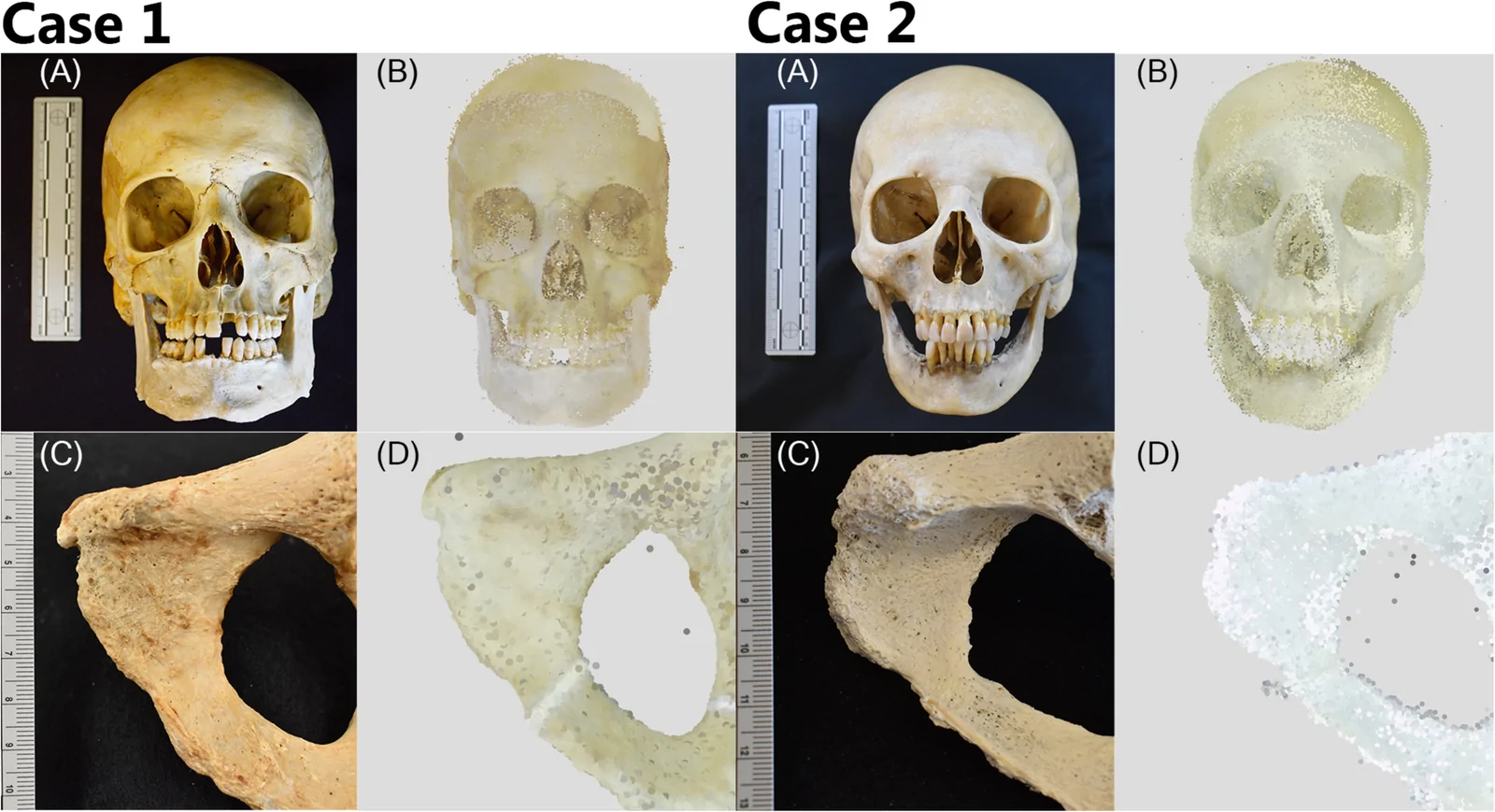
Photographs and virtual renderings of the skeletal cases. Cases are separated by panels. **A** Anterior view of the skull; **B** Virtual rendering of the skull in anterior view; **C** Ventral view of the left pubis; and **D** Virtual rendering of the left pubis in ventral view

During the rendering phase, the scanned elements had the background removed to allow for 360-degree rotation. The models were then re-positioned to the standardized anatomical position for visual assessment. Processed scans were exported as .ply files and imported into MeshLab [21] for post-processing, including removal of duplicate and outlier vertices, and merging of close vertices. The resulting 3D point-cloud models were uploaded to SketchFab (https://sketchfab.com/*)*, an online platform that allows for visualization and manipulation of 3D models. A direct visual comparison of the physical elements and rendered models is presented in Fig. 1. Point-cloud model quality statistics are presented in Table 1. All models have average point spacing of less than 0.59 mm, showing a sub-millimeter sampling density and good scan resolution. Notably, all models show right-skewed spacing distributions, suggesting points were more sparsely distributed in some areas.

**Table 1:** Point-cloud model quality statistics

Skeletal element	Model statistics	Case 1	Case 2
Skull	Bound box dimensions ($\:x\times\:y\times\:z$) (mm)	$\:199.5\times\:148.6\times\:232.3$	$\:159.7\times\:208.9\times\:183.1$
	Total points	462,175	302,536
	Average point spacing (mm)	0.43	0.59
	Median point spacing (mm)	0.37	0.52
Innominate	Bound box dimensions ($\:x\times\:y\times\:z$) (mm)	$\:129.5\times\:196.4\times\:218.6$	$\:106.6\times\:182.8\times\:205.8$
	Total points	268,932	92,073
	Average point spacing (mm)	0.23	0.49
	Median point spacing (mm)	0.22	0.37

A two-phase data collection design was implemented. For both phases, participants were asked to assess nonmetric traits used for skeletal sex [22], age estimation [23, 24], and population affinity [25, 26] estimation for the two cases. Reference descriptions along with diagrams or reference figures were provided to assist participants with trait assessment. For the first phase, a paper-based survey was administered with physical copies of the reference materials. Participants were asked to visually assess the skeletal elements, record nonmetric trait scores, and answer a series of demographic questions including years of professional experience (inclusive of schooling), number of skulls and innominates assessed prior to this study, and highest degree of education obtained. For the second phase, a digital survey was developed using Qualtrics XM (Provo, UT) survey platform. 3D point-cloud models from SketchFab, reference descriptions, and reference diagrams and/or figures were embedded directly within the survey and paired with each corresponding nonmetric trait question. The washout period between the first and second phase to minimize bias varied between six and nine weeks. For both phases, participants were permitted to omit trait scores when they felt the relevant morphology could not be assessed on the physical element (e.g., due to preservation or processing damage) or was not adequately represented in the virtual models.

Participants were recruited from the Washington Metropolitan area using the authors’ professional network. Data collection took place from June 2025 to March 2026. The data collection, processing, and analysis procedures of this study were determined to be exempt from Institutional Review Board (IRB) review by the Office of Research Integrity & Assurance of George Mason University (IRB ID: STUDY00000538). All methods were performed in accordance with the relevant guidelines and regulations.

To assess the inter-observer agreement for both physical and virtual trait scores among participants, Fleiss’ kappa was used. Inter-modality agreement, comparing agreement between physical and virtual trait scores recorded by the same observer, was assessed using Cohen’s kappa. While Cohen’s kappa was used to evaluate the degree of agreement between two raters who are classifying items into mutually exclusive categories [27], Fleiss’ kappa is an extension designed to assess agreement among more than two raters [28]. Depending on biological profile components and traits [29], Fleiss’ kappa, Fleiss’ kappa with quadratic weights, Cohen’s kappa, or Cohen’s weighted kappa with quadratic weights were calculated by skeletal element and biological profile component, as well as by individual nonmetric trait to identify traits contributing most strongly to overall agreement and/or disagreement. Specifically, weighted kappa statistics were used for ordinal traits where smaller differences in trait scores represent smaller disagreement, while greater discrepancies in trait scores represent greater disagreement. Unweighted kappa statistics were used for nominal and/or dichotomous traits, as no meaningful distance between the categories exists and disagreement in any form is treated equally [29]. All kappa statistics were interpreted according to the guidelines proposed by Landis and Koch [30]: poor (< 0.00), slight (0.00–0.20), fair (0.21–0.40), moderate (0.41–0.60), substantial (0.61–0.80), and almost perfect (0.81–1.00). Additional inter-observer agreement statistics, including two-way intraclass correlation coefficients (ICC) [31] and Gwet’s Agreement Coefficient (AC2) [32] were also estimated and are presented in the Supplementary Information, along with a discussion of why kappa was retained as the primary agreement statistic.

Nonmetric trait scores for skeletal sex were entered into MorphoPASSE [33] with random forest modeling. Trait scores were compared to U.S. reference samples of contemporary temporal period and unknown ancestry. Pubic symphysis phases were compared to the age ranges proposed by Brooks & Suchey [24] and Hartnett [23] for age estimation. Nonmetric population affinity trait scores were entered into MaMD Analytical [34] for population affinity estimation. For sex estimation, classification results with posterior probabilities greater than 0.75 were compared to the documented biological sex of the individuals, consistent with recommended posterior probability-informed thresholds [35, 36]. Results were considered accurate if the estimated skeletal sex matched the documented biological sex. For age estimation, results were considered accurate if the true age fell within the most inclusive age range, defined as the interval spanning the lowest lower bound to the highest upper bound across all applicable phase ranges, consistent with previous analytical approaches on method-derived accuracy [37, 38]. Finally, for population affinity estimation, while self-reported or perceived race does not equate population affinity categories [39], and no explicit guidelines on decision-making thresholds currently exist for nonmetric population affinity estimation, we followed the approach proposed by Hughes et al. [38], drawing on studies examining the parallel among genetic estimates of continental ancestry, self-reported racial identity, and skeletal estimates of population affinity [e.g., 40–43]. Predicted group memberships were compared to the reported racial identities of the individuals, and results were considered accurate if the estimated population affinity matched the reported race. Wilson score confidence intervals [44] were calculated for each case and biological profile component.

All statistical analyses were performed using R Studio [45] with the R Programming Language (version 4.5.2) [46] and R packages *psych* [47], *irr* [48] and *irrCAC* [49].

## Results

A total of 53 respondents participated in the first phase of data collection, while 26 completed the second phase. The demographic characteristics of the respondents are presented in Table 2. Results for inter-observer agreement among study participants for physical skeletal elements are presented in Table 3. Both cases showed “fair” agreements (kappa = 0.21–0.40) among observers for population affinity estimation (Cohen’s kappa = 0.34 and 0.28 for Case 1 and Case 2), while all other biological profile components showed only “slight” agreements (kappa = 0.00–0.20). To assess whether attrition influenced the observed agreement, Phase 1 kappa statistics were recomputed for the subset of participants who completed both phases (Table S8). Agreement estimates were largely unchanged relative to the full sample (Table 3) and exhibited similar strengths of agreement (i.e., in the same Landis and Koch [30] range).

**Table 2:** Participant demographics. A total of 53 participants completed the first phase of data collection (physical skeletal assessment), while 26 completed the second phase (virtual skeletal assessment)

		Physical assessment	Virtual assessment
Years of experience	1 to 5 years	86.79% (46)	88.46% (23)
	5 to 10 years	5.66% (3)	7.69% (2)
	10 to 15 years	1.89% (1)	3.85% (1)
	15 to 20 years	5.66% (3)	0.00% (0)
Skulls assessed	0–5	33.96% (18)	30.77% (8)
	6–10	18.87% (10)	23.08% (6)
	11–19	15.09% (8)	26.92% (7)
	20–50	20.75% (11)	15.38% (4)
	> 50	11.32% (6)	3.85% (1)
Innominate assessed	0–5	37.74% (20)	30.77% (8)
	6–10	22.64% (12)	34.62% (9)
	11–19	9.43% (5)	15.38% (4)
	20–50	16.98% (9)	11.54% (3)
	> 50	13.21% (7)	7.69% (2)

**Table 3:** Inter-observer agreement (Fleiss’ kappa) for physical skeletal assessments (*n* = 53 raters)

Biological profile component	Skeletal element	Case 1	Case 2
Skeletal sex	Skull	0.19	0.14
	Innominate	0.08	0.06
Age	Innominate	0.00	0.10^*^
Population affinity	Skull	0.34^‡^	0.28^†‡^

^*^n = 31 due to removal of incomplete observations

^†^*n* = 45 due to removal of incomplete observations

^‡^Unweighted Fleiss' kappa

Results for inter-observer agreement among study participants for virtual skeletal elements are presented in Table 4. While agreement values differed slightly compared to physical skeletal assessment, the general patterns remained similar. For both cases, population affinity estimation demonstrated “fair” agreement among observers (Cohen’s kappa = 0.30 and 0.34 for Case 1 and Case 2), while the other biological profile components showed “slight” (kappa = 0.00 –0.20) to “poor” (kappa < 0.00) agreements. Notably, the number of raters contributing to each agreement statistic ranged from 13 to 25, despite 26 respondents completing the virtual skeletal assessment survey.

**Table 4:** Inter-observer agreement (Fleiss’ kappa) for virtual skeletal assessments (*n* = 26 raters)

Biological profile component	Skeletal element	Case 1	Case 2
Skeletal sex	Skull	0.03	0.12^*^
	Innominate	0.02^†^	0.002^†^
Age	Innominate	0.01^*^	-0.02^‡^
Population affinity	Skull	0.30^§ ((^	0.34^* ((^

^*^n = 21 due to removal of incomplete observations

^†^*n* = 13 due to removal of incomplete observations

^‡^*n* = 20 due to removal of incomplete observations

^§^*n* = 25 due to removal of incomplete observations

^((^Unweighted Fleiss' kappa

**Fig. 2:**
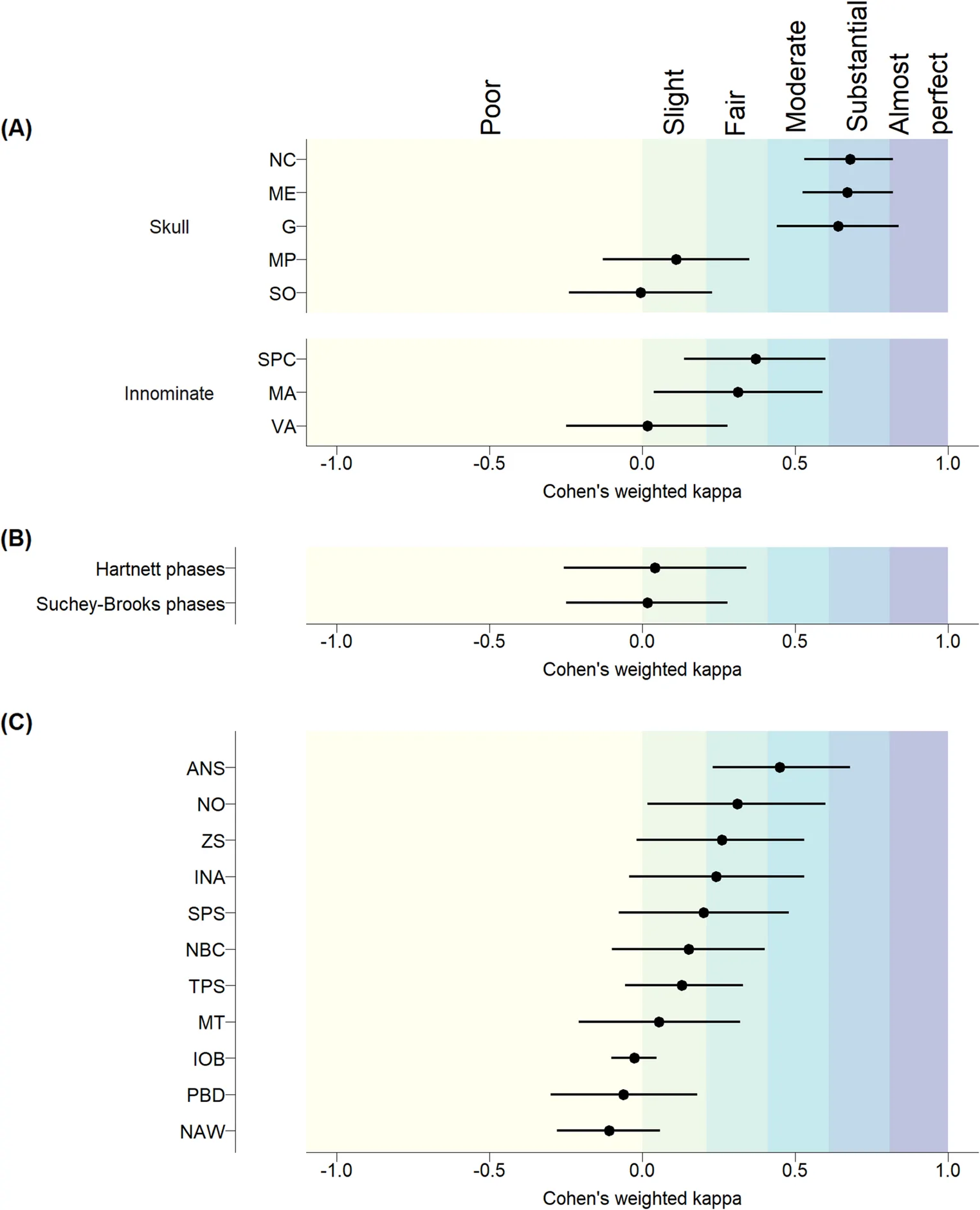
Inter-modality agreement by trait. **A** sex estimation traits of the skull and innominate, **B** age estimation (pubic symphysis) phase methods, and (**C**) cranial macromorphoscopic traits used in population affinity estimation. Points are Cohen’s weighted kappa estimates and horizontal bars are 95% confidence intervals, pooled across all raters and both cases. Background shading denotes the agreement strata of Landis and Koch (1977) [30]. Trait abbreviations: (**A**) NC, nuchal crest; ME, mental eminence; G, glabella; MP, mastoid process; SO, supraorbital margin; SPC, subpubic contour; MA, medial aspect of the ischiopubic ramus; VA, ventral arc. **C** ANS, anterior nasal spine; NO, nasal overgrowth; ZS, zygomaticomaxillary suture; INA, inferior nasal aperture; SPS, supranasal suture; NBC, nasal bone contour; TPS, transverse palatine suture; MT, malar tubercle; IOB, interorbital breadth; PBD, post-bregmatic depression; NAW, nasal aperture width

Table 5 presents the inter-modality agreement between physical and virtual trait scores recorded by the same observer. For both cases, Cohen’s kappa indicated that sex traits of the skull (0.25 and 0.16 for Case 1 and Case 2, respectively) and population affinity traits (0.23) exhibited the strongest correspondence between modalities, although agreement reached only “fair” levels. The lowest levels of agreement were observed for innominate sex traits (Cohen’s weighted kappa = 0.17 and < 0.00 for Case 1 and Case 2) and the pubic symphyseal age indicators (Cohen’s weighted kappa = 0.10 and 0.08 for Case 1 and Case 2). Inter-modality agreement for individual traits is presented in Fig. 2. Across all assessments, the highest agreement between physical and virtual observations (“substantial” agreement, kappa = 0.61–0.80) was observed for three skull sex traits (nuchal crest [Cohen’s weighted kappa = 0.68], mental eminence [Cohen’s weighted kappa = 0.67], and glabella [Cohen’s weighted kappa = 0.64]). All other sex traits showed only “fair” to “slight” agreement. Agreement for pubic symphyseal traits was also “slight.” Among population affinity traits, the anterior nasal spine (Cohen’s weighted kappa = 0.45) demonstrated “moderate” agreement (kappa = 0.41–0.60), while all other traits showed “poor” to “fair” agreement.

**Table 5:** Inter-modality agreement (Cohen’s kappa) (*n* = 26 raters)

Biological profile component	Skeletal element	Case 1	Case 2
Skeletal sex	Skull	0.25^*^	0.16^*^
	Innominate	0.17^*^	-0.02^*^
Age	Innominate	0.10^*^	0.08^*^
Population affinity	Skull	0.23	0.23

^*^Cohen’s weighted kappa with quadratic weights

Inter-observer agreement stratified by experience (novice: 10 elements or less assessed; experienced: more than 10 elements assessed, Fig. S1) showed no consistent improvement in agreement with greater experience in either modality. Note that this comparison is exploratory and may be underpowered, as the sample was heavily skewed toward early-career raters (87% reported ≤ 5 years of experience, Table 1), resulting in lower sample size for the experienced cohort across elements, biological profile components, and modalities to support a reliable contrast.

**Fig. 3:**
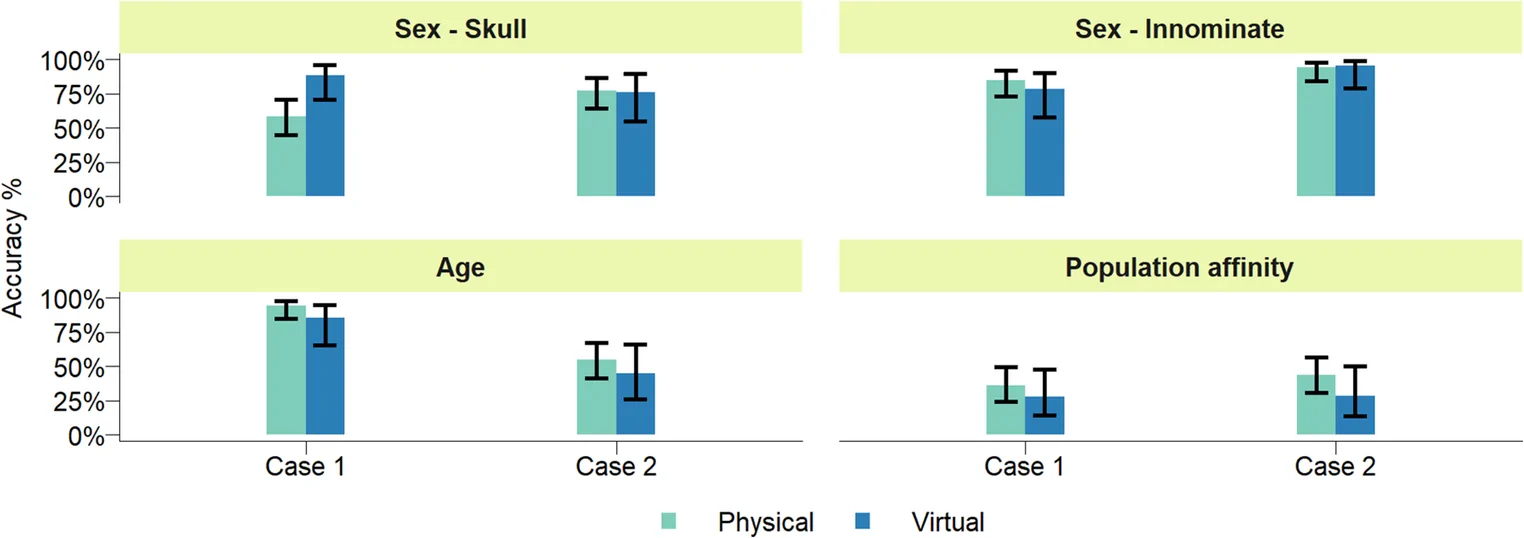
Overall accuracy (%) of biological profile estimates for Case 1 and Case 2 based on physical and virtual skeletal assessments. Results are shown for sex estimation from the skull and innominate, age estimation, and population affinity estimation. Green bars indicate physical assessments and blue bars indicate virtual assessments. Error bars represent 95% Wilson binomial confidence intervals

Figure 3 shows the classification accuracies of biological profile estimates for the two cases by assessment modalities (Table S8 and S9). In general, point estimates of accuracy for virtual assessments were lower than physical assessments across most biological profile components,, although 95% confidence intervals were overlapping in all cases. However, for skeletal sex estimation, virtual assessments produced comparable or slightly higher accuracies in some cases. Differences between modalities were more pronounced for age estimation and population affinity estimation. Note these values represent classification accuracy within this two-case sample rather than generalized method performance.

## Discussion

In this study, we investigated whether handheld laser scan-derived 3D point-cloud models can reproduce nonmetric skeletal assessment using two skeletal cases. Overall, in this sample, we found that inter-observer agreement was lower for virtual skeletal assessments than for physical skeletal assessment, and inter-modality agreements for nonmetric traits were fair at best. However, the disagreements between modalities did not substantially affect the overall sex estimation accuracy. In contrast, while nonmetric population affinity traits showed fair inter-modality agreement, virtual assessments produced lower classification accuracies. While exploratory in nature, these findings indicate that handheld laser scan-derived 3D models do not consistently reproduce the trait observations obtained from physical skeletal elements and should therefore be used with caution for nonmetric biological profile estimation.

We found that inter-observer agreement for sex estimation using nonmetric traits was low across both the skull and innominate, in both physical and virtual assessments. Despite this, the low levels of agreement did not translate into reduced classification accuracy for skeletal sex estimation within this two-case sample. While the innominate is generally more sexually dimorphic than the skull [50], the specific traits used for sex estimation were often not scorable in virtual modalities and, even under optimal conditions, showed only moderate inter-observer and modality agreement [51]. In contrast, Del Bove et al. [52] showed that within the cranium, the frontal region (glabella), and the mastoid processes are the most sexually dimorphic regions. The projection, contour, and overall robustness of these skull traits likely made them easier to assess in virtual settings, which may contribute to their comparatively elevated inter-modality agreement.

For age estimation, inter-observer agreement for pubic symphysis was low across both conditions, and inter-modality agreement was similarly low, suggesting that the pubic symphysis was difficult to assess reliably regardless of modalities. Two sets of factors likely contributed to this. First, complex and highly detailed phase-based descriptions [23, 24] allow raters to prioritize different features, thus lowering inter-observer consistency [53]. This effect could be compounded by a participant pool composed predominantly of students and trainees, especially for the second phase of the study. However, it should be noted that the present data could not isolate an experience effect. Second, similar to digitally-derived and 3D-printed proxies [54], such models often demonstrated limited ability to capture textured minutiae and subtle details of the symphyseal phase, and 3D rendering could smooth or omit details, resulting in “loss of bone” in virtual modalities, as reported even for CT-derived models [55]. This is especially true for less experienced observers working from virtual modalities [54, 56, 57]. Even when intra- and inter-observer error in the scans themselves is negligible, observer-level error during assessment remains a source of discrepancy [14].

Notably, the inability to capture finer skeletal details, as observed in this study for nonmetric innominate sex traits and the pubic symphysis, is not likely due to poor scan resolution, as point-cloud model quality statistics clearly demonstrated sub-millimeter sampling density well below the typical osteometric error thresholds of 2 mm [11, 58]. Rather, the point-cloud representations generated by laser scanning lack the shading and surface continuity that convey subtle textural and relief characteristics, which are central to assessing these traits. This is consistent with the observation that surfaced-mesh rendering from CT scans assessed by Colman et al. [10] showed comparatively higher inter-observer agreement when assessing the Klales et al. traits [59].

Finally, population affinity traits showed fair inter-observer and inter-modality agreement overall, while classification accuracy remained low across all conditions. Population affinity traits encompass a variety of traits and use a more variable and complex scoring system that includes ordinal, nominal, and dichotomous traits, which introduces multiple sources of measurement variability. Moreover, observers may gravitate toward different subsets of traits [29], further contributing to trait-level variability. Some population affinity traits are also more tactile [29], and the loss of tactility has been identified as a source of increased uncertainty during trait assessment in virtual modalities [60]. Critically, the fair agreement for population affinity did not translate into accurate classification in the present two-case sample, underscoring that reliability and validity are distinct. While the estimated group affiliation often cannot translate directly into self-reported or perceived social race categories [43], a previous study using a large identified case sample from across the United States found less than 1% of the decedents’ self-reported race and/or ethnicity categories deviated from categories commonly used in forensic anthropology casework or other forensic contexts (i.e., NamUs categories) [38]. Because of this, social race categories served as a practical reference standard here. The population affinity classification accuracies reported here should therefore be interpreted as an imperfect but informative indication of how the practice of population affinity estimation translates into virtual settings. However, as with the other components, this interpretation is limited by the two-case design.

The present study relied on a single pool of participants to respond and record their assessments on two different modalities across two time points. Survey attrition [61], the noticeable dropout of participants between the first and second phase likely limited the statistical power of the study [62, 63]. However, when limiting the sample to individuals who completed both phases of data collection, agreement statistics for physical assessment (the first phase) remained similar, suggesting that the observed agreement values were not substantially affected by attrition. Nonetheless, the reduced sample size may lead to imprecise estimates and therefore should be interpreted with caution.

In addition, while the majority of the participants in this study were considered early career/less experienced, we found no consistent improvement in agreement with greater experience in either modality, though the small number of experienced participants limits this comparison. This is consistent with previous studies showing dry-bone osteological experience alone is not predictive of measurement error or discrepancy in virtual settings [3, 60, 64–66]. It is likely that a combination of both dry-bone and virtual experience underlies observers’ ability to effectively and accurately assess 3D models [67, 68]. Moreover, while a washout period between assessments, exceeding the recommended minimum of 2 weeks used in proficiency testing such as the College of American Pathologists [69, 70], was implemented, it is likely that recall bias, where participants remembered some traits scored on the physical skeletal elements, still persists. A previous study showed participants were able to recall approximately 30% of the cases previously seen following a four-week interval [71], suggesting some inter-modality agreement may be confounded by memory effects. However, given the relatively low inter-modality agreement statistics, the effects could be considered minimal. Nonetheless, the results of the study provided an early benchmark for assessing the suitability of handheld 3D scanning technologies in forensic anthropological practice.

Previous studies suggested that CT-derived models demonstrated “moderate” to “almost perfect” agreement with dry bone observations for non-metric trait assessment [10], showing that CT is a suitable modality for virtual non-metric skeletal analysis. However, such technology may not be readily available to practitioners. For example, in the United States, fewer than 10% of the medical examiner and coroner offices reported having direct access to CT imaging systems [72]. In contrast, 3D laser scanners are becoming increasingly common within forensic and investigative settings [73], and this accessibility makes the findings of the current study particularly relevant to the forensic science community. Our results showed a widely available handheld scanner can support some aspects of nonmetric biological profile assessments. Although agreement declined relative to physical skeletal assessment, classification accuracy was more variable, remaining comparable for nonmetric sex estimation. Importantly, previous evaluations of laser-scanned skeletal elements have primarily focused on metric consistency [16, 19]. To our knowledge, this is the first study to systematically investigate a 3D handheld laser scanner on non-metric biological profile estimation using a multi-observer, inter-modality design. Future validation studies using more diverse case examples and a larger sample size are warranted to further investigate the general applicability of handheld laser scanning for nonmetric skeletal assessment.

## Conclusion

This study indicates that handheld laser scanning, commonly available in forensic and investigative settings, is a promising but not interchangeable alternative to physical examination for nonmetric biological profile estimation: it preserved accuracy for shape-driven assessments such as cranial sex estimation while showing reduced agreement for traits dependent on fine surface features, suggesting that its utility is trait-dependent. For training and data-sharing applications (e.g., in research or casework settings), these results suggest that point-cloud models may support instruction and remote assessment for shape-driven traits, while traits dependent on finer surface details may require higher-precision imaging modalities or access to physical specimens. The routine adoption of such technology, however, warrants validation using more diverse case examples in larger samples.

## Supplementary Information

Below is the link to the electronic supplementary material.

Supplementary Material 1

## Data Availability

Data were deposited into the George Mason University Dataverse and are available at the following URL: https://doi.org/10.13021/ORC2020/HICSJ0.
