# Supplementary material for: Applicability of handheld laser scanning for nonmetric skeletal assessment in biological profile estimation: a preliminary two-case study
Source: Int J Legal Med. 2026 Aug 4;140(6):4307–17. doi: 10.1007/s00414-026-03943-6 (PMC13658533; doi:10.1007/s00414-026-03943-6)
Supplement: Supplementary file 1 [file 414_2026_3943_MOESM1_ESM.pdf]

Supplemental Information for *Applicability of Handheld Laser Scanning for Nonmetric Skeletal Assessment in Biological Profile Estimation: A Preliminary Two-Case Study*

Effects of participant experience

Figure S1. Inter-observer agreement stratified by participant experience.

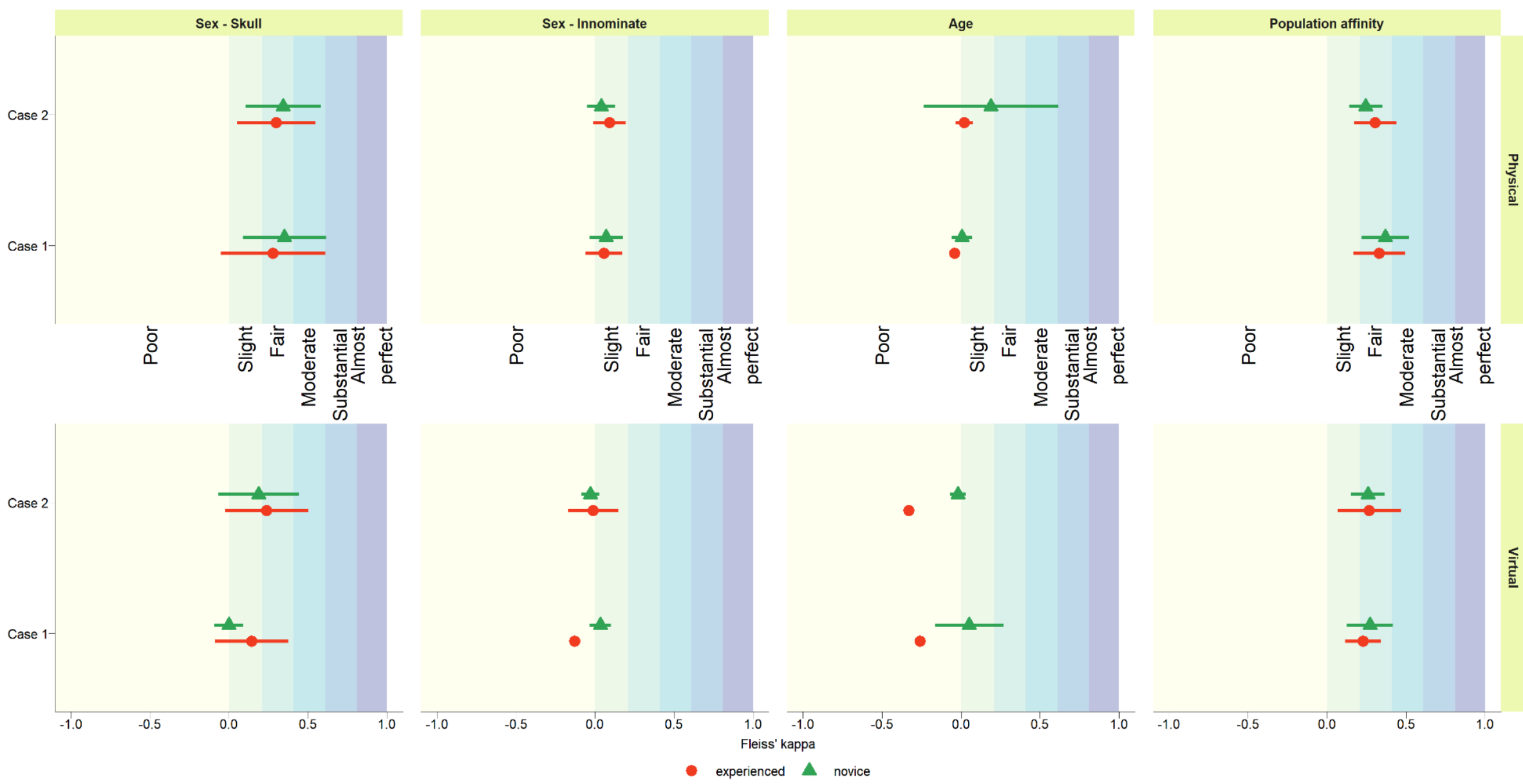

Reference trait scores

Table S1. The nonmetric trait scores, nonmetric sex and population affinity estimation results for the cases included in the current study. All nonmetric traits were scored independently by two observers according to the scoring guidelines and manuals referenced in the main text. The interobserver agreements for the nonmetric trait scores reached “substantial” to “almost perfect” agreement across all biological profile components (Cohen’s weighted kappa for skeletal sex, age, and population affinity traits = 0.8, 0.64, and 0.89, respectively), notably higher than the interobserver agreement among study participants (main text), consistent with the reference scorers' greater experience.

**Nonmetric sex traits**	**Case 1**	**Case 2**
Glabella (G)	4	1
Nuchal crest (NC)	4	1
Mental eminence (ME)	4	2
Supraorbital margins (SO)	3	1
Mastoid process (MP)	4	4
Ventral arc (VA)	5	2
Subpubic concavity (SPC)	4	2
Medial aspect of ischio-pubic ramus (MA)	4	1
**Skeletal sex estimation results**		
MorphoPASSE (Klales, 2018)<br>(posterior probability / model accuracy)	Male<br>PP: > 0.999<br>92.00%	Female<br>PP: 0.946<br>92.00%
**Pubic symphysis phases**		
Brooks & Suchey (1990)	3	6
Hartnett (2010)	3	7
**Cranial macromorphoscopic traits**		
Anterior nasal spine (ANS)	3	2
Inferior nasal aperture (INA)	5	4
Interorbital breadth (IOB)	1	2
Malar tubercle (MT)	2	0
Nasal aperture shape (NAS)	2	1
Nasal aperture width (NAW)	1	2
Nasal bone contour (NBC)	4	1

Nasal bone shape (NBS)	2	2
Nasal overgrowth (NO)	0	0
Nasofrontal suture (NFS)	3	1
Orbital shape (OBS)	3	1
Postbregmatic depression (PDB)	0	0
Posterior zygomatic tubercle (PZT)	1	2
Supranasal suture (SPS)	1	0
Transverse palatine suture (TPS)	3	3
Palate shape (PS)	3	-
Zygomaticomaxillary suture course (ZS)	0	-
**Population affinity estimation results**		
MaMD Analytical 1.1.0<br>(posterior probability / model accuracy)	Euro-American<br>PP: 0.537<br>62.22%	American Indian<br>PP: 0.808<br>72.50%

Agreement statistics

Intraclass correlation coefficients (ICC) are descriptive statistics that assess units organized into groups and assist with determining how strongly units in the same group resemble each other (Koo et al., 2016). While it is viewed as a type of correlation, it differs from other correlation measures, as it operates on data structured as groups rather than data structured as paired observations. In the case of the current study, ICC can be appropriate because each biological profile component of each case is rated by a distinct set of raters randomly chosen from a larger population of possible respondents, on an ordinal scale with an underlying continuous trait expression (Klales et al., 2020; Lesciotto & Doershuk, 2018; Shirley & Ramirez Montes, 2015; Spiros et al., 2023). However, ICC treats trait scores as quantitative/continuous and estimates the proportion of total variance attributable to systematic differences among traits. It reflects *how well raters rank/track the traits* rather than categorical agreement among raters. Therefore, the values may be **inflated**. Because categorical *agreement* among raters is the more appropriate measure for nonmetric traits, the following ICC values were used primarily to demonstrate the *patterns* of agreement across biological profile components and skeletal elements, complementary to the kappa statistics reported in the main text.

Table S2. Inter-observer agreement (ICC) for physical skeletal assessments (n = 53 raters).

**Biological profile component**	**Skeletal element**	**Case 1**	**Case 2**
Skeletal sex	Skull	0.96	0.97
	Innominate	0.85	0.83
Age	Innominate	0.52	0.89*
Population affinity	Skull	0.99	0.99

*n = 31 due to removal of incomplete observations.

Table S3. Inter-observer agreement (ICC) for virtual skeletal assessments (n = 26 raters).

**Biological profile component**	**Skeletal element**	**Case 1**	**Case 2**
Skeletal sex	Skull	0.72*	0.87§
	Innominate	0.46†	0.46§
Age	Innominate	0.91‡	0.46†
Population affinity	Skull	0.96*	0.95§

*n = 24 due to removal of incomplete observations.
†n = 20 due to removal of incomplete observations.
‡n = 18 due to removal of incomplete observations.
§n = 21 due to removal of incomplete observations.

Additionally, for nonmetric traits, some researchers may prefer interobserver reliability statistics other than ICC, as some nonmetric variables can be considered nominal instead of ordinal, potentially violating assumptions underlying ICC analyses (Liebenberg et al., 2025). To further assess interobserver reliability, **average Cohen's weighted kappa** (Light, 1971; McHugh, 2012), and **weighted Gwet's Agreement Coefficient (AC2)** (Gwet, 2008) were also calculated alongside ICC. Both these statistics aim to compare the observed agreement rate with fellow assessors, but they operate differently depending on the prevalence structure and assumptions of independence between raters. Cohen's kappa considers that agreement could happen by chance, which leads to a more accurate reflection of interobserver reliability. This kappa statistic ranges from -1 to +1 (McHugh, 2012). Average Cohen's weighted kappa (Light's kappa; Light, 1971) preserves the original pairwise kappa framework (Xie et al., 2017) and is relatively unbiased compared to other multi-rater statistics (Sertdemir et al., 2013). Average (Light's) Cohen's weighted kappa is reported alongside Fleiss' kappa (main text) to show that the agreement pattern is not an artifact of a single kappa formulation.

TABLE S4. Inter-observer agreement (average Cohen's weighted κ) for physical skeletal assessments (n = 53 raters).

**Biological profile component**	**Skeletal element**	**Case 1**	**Case 2**
Skeletal sex	Skull	0.32	0.32
	Innominate	0.10	0.07
Age	Innominate	0.03	0.24*
Population affinity	Skull	0.67	0.51†

*n = 31 due to removal of incomplete observations.
†n = 45 due to removal of incomplete observations.

TABLE S5. Inter-observer agreement (average Cohen's weighted κ) for virtual skeletal assessments (n = 26 raters).

**Biological profile component**	**Skeletal element**	**Case 1**	**Case 2**
Skeletal sex	Skull	0.06	0.29
	Innominate	0.44	0.39
Age	Innominate	0.04	0.04
Population affinity	Skull	0.04	0.03

Researchers may also prefer AC2 over average Cohen's weighted kappa for assessing binary classification, particularly in cases where prevalence in one statistical sampling category is high, which may lead to unstable kappa values (Gwet, 2008). While AC2 is prevalence-robust, Cohen's kappa and Fleiss' kappa are more commonly used in biological and forensic anthropology, making them more comparable across studies. Here, AC2 is reported again to demonstrate the *patterns* of agreement across biological profile components and skeletal elements, complementary to the kappa statistics reported in the main text.

TABLE S6. Inter-observer agreement (Gwet's AC2) for physical skeletal assessments (n = 53 raters).

**Biological profile component**	**Skeletal element**	**Case 1**	**Case 2**
Skeletal sex	Skull	0.31	0.31
	Innominate	0.27	0.24
Age	Innominate	0.19	0.14*
Population affinity	Skull	0.43	0.33

*n =31 due to removal of incomplete observations.

TABLE S7. Inter-observer agreement (Gwet's AC2) for virtual skeletal assessments (n = 26 raters).

**Biological profile component**	**Skeletal element**	**Case 1**	**Case 2**
Skeletal sex	Skull	0.16	0.26
	Innominate	0.02	0.07
Age	Innominate	0.08	-0.03
Population affinity	Skull	0.31	0.37

Taken together, the convergence in overall pattern across agreement statistics indicates the results are not a statistical artifact but rather the actual patterns of agreement across biological profile components.

TABLE S8. Inter-observer agreement (Fleiss' kappa) for physical skeletal assessments among the subset of participants who completed both phases. Values are provided for comparison with the full-sample physical assessment agreement (Table 3) to assess the influence of attrition on observed agreement.

**Biological profile component**	**Skeletal element**	**Case 1**	**Case 2**
Skeletal sex	Skull	0.18*	0.16+
	Innominate	0.08‡	0.05‡
Age	Innominate	-0.02+	0.16§
Population affinity	Skull	0.31((¶	0.29+ ¶

*n = 26; +n = 21; ‡n = 23; §n = 20; ((n = 25.

¶ Unweighted Cohen's kappa.

<u>Classification accuracy results</u>

TABLE S9. Accuracies for physical skeletal assessments (n = 53 raters).

**Biological profile component**	**Skeletal element**	**Case 1**	**Case 2**
Skeletal sex	Skull	58.49%	77.36%
	Innominate	84.91%	94.34%
Age	Innominate	94.34%	54.72%
Population affinity	Skull	35.85%	43.40%

TABLE S10. Accuracies for virtual skeletal assessments.

**Biological profile component**	**Skeletal element**	**Case 1**	**Case 2**
Skeletal sex	Skull	88.46%*	76.19%+
	Innominate	78.26%‡	95.65%‡
Age	Innominate	85.71%+	45.00%§
Population affinity	Skull	28.00%((	28.57%+

*n = 26; +n = 21; ‡n = 23; §n = 20; ((n = 25.

References

Brooks, S., & Suchey, J. M. (1990). Skeletal age determination based on the os pubis: A comparison of the Acsádi-Nemeskéri and Suchey-Brooks methods. *Human Evolution*, *5*(3), 227–238. https://doi.org/10.1007/BF02437238

Gwet, K. L. (2008). Computing inter-rater reliability and its variance in the presence of high agreement. *British Journal of Mathematical and Statistical Psychology*, *61*(1), 29–48. https://doi.org/10.1348/000711006X126600

Hartnett, K. M. (2010). Analysis of Age-at-Death Estimation Using Data from a New, Modern Autopsy Sample—Part I: Pubic Bone*,[†]. *Journal of Forensic Sciences*, *55*(5), 1145–1151. https://doi.org/10.1111/j.1556-4029.2010.01399.x

Klales, A. R. (2018). *MorphoPASSE: the Morphological Pelvis and Skull Sex Estimation Database* (Version 1.0). Washburn University. https://www.morphopasse.com/program.html

Klales, A. R., Garvin, H., Gocha, T. P., Lesciotto, K. M., & Walls, M. (2020). Examining the Reliability of Morphological Traits for Sex Estimation Implications for the Walker (2008) and Klales et al. (2012) Methods. *Forensic Anthropology*, *3*(3), 139–150. https://doi.org/10.5744/fa.2020.4015

Koo, T. K., Li, M. Y., Koo, T. K., & Li, M. Y. (2016). A Guideline of Selecting and Reporting Intraclass Correlation Coefficients for Reliability Research. *Journal of Chiropractic Medicine*, *15*(2), 155–163. https://doi.org/10.1016/J.JCM.2016.02.012

Lesciotto, K. M., & Doershuk, L. J. (2018). Accuracy and Reliability of the Klales et al. (2012) Morphoscopic Pelvic Sexing Method. *Journal of Forensic Sciences*, *63*(1), 214–220. https://doi.org/10.1111/1556-4029.13501;SUBPAGE:STRING:FULL

Liebenberg, L., Stull, K. E., & L'Abbé, E. N. (2025). Patterns of observer error in scoring macromorphoscopic traits for population affinity. *Journal of Forensic Sciences*, *70*(4), 1489–1500. https://doi.org/10.1111/1556-4029.70063

Light, R. J. (1971). Measures of response agreement for qualitative data: Some generalizations and alternatives. *Psychological Bulletin*, *76*(5), 365. https://doi.org/10.1037/H0031643

McHugh, M. L. (2012). Interrater reliability: the kappa statistic. *Biochemia Medica*, *22*(3), 276–282.

Sertdemir, Y., Burgut, H. R., Alparslan, Z. N., Unal, I., & Gunasti, S. (2013). Comparing the methods of measuring multi-rater agreement on an ordinal rating scale: A simulation study with an application to real data. *Journal of Applied Statistics*, *40*(7), 1506–1519. https://doi.org/10.1080/02664763.2013.788617

Shirley, N. R., & Ramirez Montes, P. A. (2015). Age estimation in forensic anthropology: Quantification of observer error in phase versus component-based methods. *Journal of Forensic Sciences*, *60*(1), 107–111. https://doi.org/10.1111/1556-4029.12617

Spiros, M. C., Nakhaeizadeh, S., Thompson, T. J. U., Morgan, R. M., Arvidsson, M., Olsson, V., Berivoe, A., & Hefner, J. T. (2023). Using Eye-Tracking Technology to Quantify the Effect of Experience and Education on Forensic Anthropological Analyses. *Forensic Anthropology*, *6*(1), 25–35. https://doi.org/10.5744/fa.2022.0001

Xie, Z., Gadepalli, C., & Cheetham, B. M. G. (2017). REFORMULATION AND GENERALISATION OF THE COHEN AND FLEISS KAPPAS. *LIFE: International Journal of Health and Life-Sciences*, *3*(3), 01–15. https://doi.org/10.20319/LIJHLS.2017.33.115
